# The association of mindfulness with athletes’ fear of failure: the mediating roles of perfectionism and ego-depletion

**DOI:** 10.3389/fpubh.2025.1643131

**Published:** 2025-09-23

**Authors:** Zhangyi Zhong, Hongyu Jiang, Huilin Wang

**Affiliations:** ^1^School of Physical Education, Hunan University of Science and Technology, Xiangtan, China; ^2^School of Business, Hunan University of Science and Technology, Xiangtan, China

**Keywords:** mindfulness, fear of failure, perfectionism, ego-depletion, athletes

## Abstract

**Introduction:**

Failure is an inevitable part of an athlete’s career, often bringing emotional setbacks that may affect performance and long-term development. This study adopted a cross-sectional design using convenience and snowball sampling to explore how mindfulness relates to athletes’ fear of failure.

**Methods:**

A total of 285 valid responses were gathered from university athletes, youth training centers, and sports schools across Hunan, Hubei, and Sichuan provinces. Structural equation modelling (AMOS v26) was employed to test the proposed model.

**Results:**

The findings indicated that mindfulness was negatively associated with perfectionism and ego-depletion. In contrast, both perfectionism and ego-depletion were positively related to fear of failure—that is, individuals with higher levels of perfectionism experienced greater ego-depletion. Furthermore, both perfectionism and ego-depletion mediated the relationship between mindfulness and fear of failure. These results suggest that athletes with higher levels of mindfulness tend to exhibit lower levels of perfectionistic tendencies and ego-depletion, and consequently experience less fear of failure.

**Conclusion:**

Based on these findings, it is recommended that mindfulness training be incorporated into regular athletic programs, supported by adequate resources to promote athletes’ mental well-being and competitive resilience. Coaches and family members should adopt more supportive and encouraging attitudes, reducing the psychological harm athletes may experience after failure. Moreover, athletes should acknowledge and accept their fear of failure, and seek professional support in a timely manner when troubled by negative psychological states, to ensure both physical and mental well-being.

## 1 Introduction

Every athlete encounters failure at different stages of their sporting career, experiencing the pain associated with such setbacks. How athletes cope with failure significantly influences both their performance outcomes and long-term career development (43). Following a defeat, athletes often experience intense anxiety and regret over their performance and results, while becoming the focus of criticism from both their immediate surroundings and social media (19). This can trap them in extreme negative emotions. After experiencing the discomfort associated with failure, athletes may develop a fear of failure, striving to avoid such outcomes in their future sporting careers (47).

The fear of failure is primarily driven by anticipated shame and the avoidance of potential negative consequences. According to Gustafsson et al. (24), when facing failure, individuals first experience shame and embarrassment, followed by feelings of uncertainty about the future, loss of future opportunities, concerns about diminished social value and influence, and fears of disappointing important others (e.g., parents, partners, coaches), among these, concerns regarding social and self-value have the most profound impact on athletes (8, 50).

Research has suggested that a moderate level of fear of failure can enhance athletes’ commitment to training and competition, thereby improving performance (23, 31). As competition intensifies and victories become harder to secure, athletes are increasingly exposed to repeated setbacks. Coupled with physical fatigue from rigorous training, this often results in highly unstable performance (37). Notably, research indicates that the peak years for elite athletic performance often align with the highest risk period for the onset of mental health disorders (37). Many sports reach peak performance levels at relatively young ages, when athletes have yet to develop mature value systems and cognitive frameworks, making it difficult for them to cope with stressors that even mature individuals may struggle to handle (2).

Recent evidence from Chinese elite athletes during the post-COVID-19 period highlights this concern. For instance, a cross-sectional study conducted in August 2021 among Chinese winter-sport elite athletes preparing for the Beijing 2022 Winter Olympics reported prevalence rates of 20.1% for depressive symptoms and 15.0% for anxiety symptoms, alongside 13.0% reporting insomnia and 3.5% having suicidal ideation (27). Additionally, nearly 35% of elite team sport athletes have experienced psychological disorders such as depression, anxiety, and burnout (5, 36), exposing athletes to significant physical and mental health risks. If athletes are unable to regulate their emotions and continue to suffer from the fear of failure, it can lead to performance declines, hinder the development of their potential and skills, and even obstruct their future achievements (53). Therefore, there is an urgent need for a psychological skill that can help them regulate their negative emotions.

Mindfulness, a widely used tool in psychology and health fields, is considered one of the most effective interventions for emotion regulation and alleviating negative mental states (55). Mindfulness is a seemingly simple psychological skill that connects an individual’s various experiences, helping to reduce suffering and accumulate psychological resources for positive change (45). It emphasizes focused attention on present tasks while disregarding distractions, which is a crucial prerequisite for successful athletic performance (13, 32). It has also been shown to reduce stress, ease negative emotions and physical pain, and enhance overall well-being and a sense of accomplishment (21). Mindfulness practice strengthens the connection between the body and mind, improving awareness of physiological and psychological states such as tension, fear, thirst, and fatigue (38). When individuals become aware of their internal states or current situations, mindfulness enables them to detach from emotional reactions and respond to circumstances with a non-judgmental attitude (6).

In addition, studies have shown that perfectionism and ego-depletion are important factors contributing to heightened fear-related emotions among athletes (9, 12, 46). Perfectionism is traditionally viewed as a stable personality trait and is widely recognized as a significant contributor to a range of psychological difficulties. Hollender (26) defined perfectionism as the tendency to demand performance from oneself or others beyond what is realistically required, with the degree of such demands highly correlated with the individual’s level of perfectionism. In simple terms, perfectionists harshly criticize themselves or others for failing to meet standards, and even when standards are met, they tend to reframe them as insufficiently high (44). Empirical studies have established a close link between perfectionism and fear of failure. For instance, Frost and Henderson (17) found a strong positive correlation between the two, while Stoeber and Becker (46) demonstrated that perfectionism is more strongly associated with fear of mistakes and failure than with the pursuit of success. This suggests that perfectionism aligns more closely with avoidance motivation. Further, Sagar and Stoeber (41) noted that perfectionists who base their self-worth on performance outcomes tend to engage in harsh self-evaluation, increasing their vulnerability to shame and guilt, which in turn amplifies fear of failure and reduces the ability to experience satisfaction from success.

One of the core constructs in this study is ego-depletion, a psychological state that has received increasing attention in self-regulation research. Ego-depletion refers to a temporary reduction in psychological and physical resources resulting from the repeated exertion of self-control (4). The prevailing view holds that self-control relies on limited internal resources (29). According to the Strength Model of Self-Control, deliberate and effortful actions quickly deplete these resources, resulting in a state of ego-depletion in which subsequent self-regulation becomes significantly impaired (28). However, individuals vary in the extent to which they expend self-control resources on the same task—that is, they differ in their sensitivity to resource depletion. Some individuals deplete these resources more quickly than others when performing the same task (42). Results indicated that depletion sensitivity moderates the ego-depletion effect. Specifically, individuals with higher sensitivity to self-control resource consumption performed worse on subsequent self-regulatory tasks, suggesting a stronger ego-depletion effect compared to those with lower sensitivity. This highlights the importance of considering individual differences in depletion sensitivity when examining self-control processes. Therefore, this study approaches the issue from the perspective of depletion sensitivity, focusing on individuals’ ego-depletion states by examining how stable differences related to depletion sensitivity are associated with fear of failure.

For athletes, ego-depletion can significantly impact competitive outcomes and training performance. Studies show that engaging in two consecutive self-control tasks exacerbates resource depletion, making athletes more susceptible to ego-depletion (15). In daily training and competitions, athletes are often exposed to uncontrollable factors that force frequent mental adjustments and regulation of performance. Repeated cycles of self-control accelerate ego-depletion, leading to cognitive impairments, attention lapses, decision-making errors, and, in severe cases, physical injuries (18). To deliver superior performance, athletes must overcome fear of failure and exert self-control to manage injuries, mistakes, and impulsive behaviors during competitions (18). However, the Strength Model of Self-Control posits that repeated exertion of self-regulation depletes psychological resources, thereby increasing susceptibility to ego-depletion (28). Under ego-depleted conditions, the likelihood of successful self-regulation diminishes, impairing athletic performance and potentially triggering impulsive behaviors such as severe injuries or rule violations (40). Post-competition failures further intensify feelings of shame and social evaluation concerns, reinforcing fear of failure (12).

Existing research on athletes’ fear of failure has largely centered on its links to shame (24), anxiety (22), burnout (24), and aggression (39). However, most studies on fear of failure have been conducted in academic (10, 47) and business settings (20, 52), with relatively little attention given to the outcome-focused context of sport—despite it being a key domain of personal achievement (14). Existing research has focused more on the consequences (56) and measurement of fear of failure (10), with relatively few studies investigating its antecedents or offering intervention strategies for athletes.

Unlike previous studies, this research approaches the issue from a cognitive perspective, focusing on athletes’ fear of failure and proposing mindfulness meditation as an intervention to alleviate the negative effects associated with this fear. Although mindfulness has demonstrated potential benefits in addressing perfectionism, ego-depletion, and fear of failure among athletes, our understanding of how these factors are interrelated and interact with one another remains limited. This study aims to explore the underlying mechanisms among these variables and identify effective strategies to reduce athletes’ fear of failure.

Based on the above, the following hypotheses are proposed:

*Hypothesis 1 (H1):* Mindfulness is negatively associated with perfectionism.

*Hypothesis 2 (H2):* Mindfulness is negatively associated with ego-depletion.

*Hypothesis 3 (H3):* Perfectionism is positively associated with ego-depletion.

*Hypothesis 4 (H4):* Perfectionism is positively associated with fear of failure.

*Hypothesis 5 (H5):* Ego-depletion is positively associated with fear of failure.

*Hypothesis 6 (H6):* Perfectionism and ego-depletion mediate the relationship between mindfulness and fear of failure.

**Figure 1:**
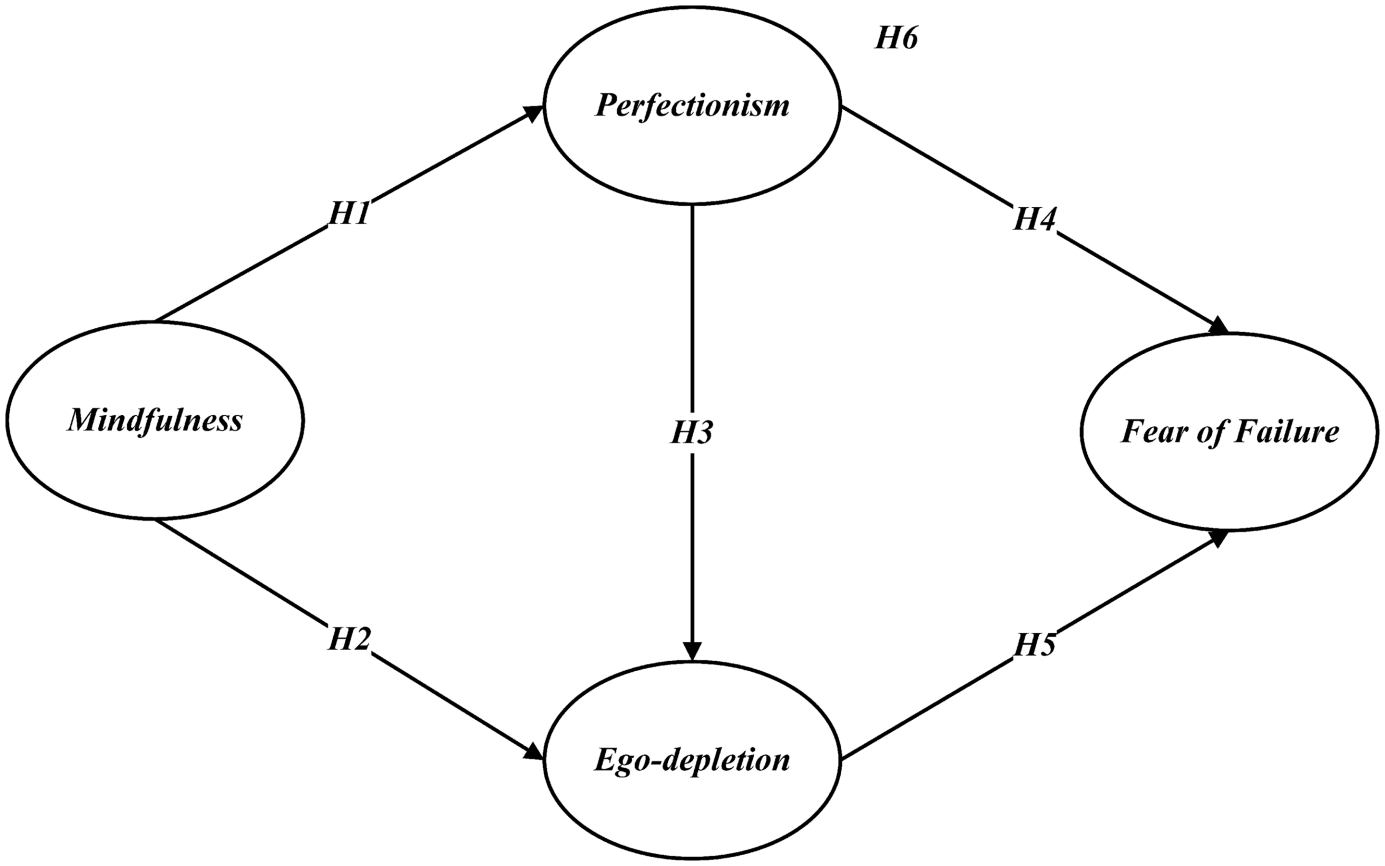
Hypothesized research model.

All hypotheses are summarized in Figure 1.

Therefore, this study aims to: (1) understand the issue of fear of failure among university athletes in China, (2) explore the associations among mindfulness, perfectionism, ego-depletion, and athletes’ fear of failure, and (3) provide intervention suggestions for both athletes and coaches to address fear of failure.

## 2 Methodology

### 2.1 Participants and pro**ced**ure

This study utilized snowball and convenience sampling to administer a questionnaire survey targeting adolescent athletes with prior training experience. Data collection took place in November and December 2024 across youth training centers, universities, and competitive sports schools in Hunan, Hubei, and Sichuan provinces. Participants were recruited via social media platforms such as WeChat, Weibo, and QQ. Athletes who expressed interest were invited to complete an online questionnaire, with informed consent obtained beforehand. As a token of appreciation, each respondent received a bottle of sports drink upon completion. Participants were also encouraged to share the questionnaire link with teammates and peers. In total, 334 questionnaires were distributed, and 285 valid responses were collected, resulting in an effective response rate of approximately 85.3%.

Table 1 presents the demographic characteristics of the sample. Key findings include: (1) a nearly equal gender distribution; (2) close to 70% of participants were aged 17–23; (3) around one-third specialized in badminton or track and field; and (4) approximately two-thirds reported experiencing 4–9 stress- or anxiety-related incidents in the past 6 months.

**Table 1:** Demographic characteristics (*N* = 285).

Profile	*n*	%
Gender		
Male	147	51.6
Female	138	48.4
Age group		
Under 17 years old	33	11.6
17–20 years old	83	29.1
20–23 years old	119	41.8
Above 23 years old	50	17.5
Sport type		
Football	19	6.7
Basketball	28	9.8
Badminton	40	14.0
Volleyball	27	9.5
Table Tennis	31	10.9
Tennis	25	8.8
Track and field	56	19.6
Gymnastics	12	4.2
Taekwondo	19	6.7
Others	28	9.8
Frequency of stress- or anxiety-related errors		
1–3 times	53	18.6
4–6 times	89	31.2
7–9 times	90	31.6
10 times or more	53	18.6

### 2.2 Instruments

The questionnaire comprised five sections. The first section collected demographic information, including gender, age, sport type, and the number of errors attributed to stress or anxiety over the past 6 months. The second section employed the Mindful Attention Awareness Scale (MAAS-5), developed by Van Dam et al. (49), which includes five items (see Table 2) to assess respondents’ level of mindfulness. The third section employed the Performance Failure Appraisal Inventory (PFAI) developed by Henry et al. (25), consisting of fifteen items to assess respondents’ fear of failure. A sample item was: “When I am failing, I blame my lack of talent.” The fourth section used the Frost Multidimensional Perfectionism Scale-Brief (F-MPS-Brief) developed by Burgess et al. (7), comprising six items to measure respondents’ levels of perfectionism. A sample item was: “I expect higher performance in my daily tasks than most people.” The fifth section used the Depletion Sensitivity Scale (DSS) developed by Salmon et al. (42), containing six items to assess respondents’ level of depletion sensitivity. A sample item was: “I have difficulties focusing my attention after I exerted a lot of mental effort.”

**Table 2:** Mindful attention awareness scale (MAAS-5).

Items
1. It seems I am “running on automatic,” without much awareness of what I’m doing. 2. I rush through activities without being really attentive to them. 3. I get so focused on the goal I want to achieve that I lose touch with what I’m doing right now to get there. 4. I do jobs or tasks automatically, without being aware of what I’m doing. 5. I find myself doing things without paying attention.

The instruments used in this study have demonstrated acceptable levels of reliability and validity. Internal consistency for each scale was assessed using Cronbach’s *α*, all of which exceeded the acceptable threshold of 0.8. Specifically, the MAAS-5 demonstrated good internal consistency (*α* = 0.930), indicating reliability in measuring mindfulness. The Multidimensional Perfectionism Scale showed strong internal consistency across subscales (*α* = 0.932). For the Depletion Sensitivity Scale, although the AVE was borderline at 0.538, composite reliability was acceptable (CR = 0.873), suggesting sufficient convergent validity. Finally, the Performance Failure Appraisal Inventory also demonstrated adequate reliability (α = 0.842). These values provide evidence for the internal validity of the instruments used in this study. These psychometric properties are also supported by our sample data (see Section 3.1 for details).

### 2.3 Data analysis

This study employed AMOS v23 to construct a structural equation model (SEM) examining how mindfulness promotes cognitive reappraisal and psychological resilience, thereby enhancing athletes’ distress tolerance. Model parameters were estimated using the maximum likelihood (ML) method, following a two-step approach: first, the reliability and validity of the measurement model were evaluated; second, model fit indices and path coefficients were assessed, including tests for mediation effects.

To address potential common method variance (CMV) associated with self-reported data, Harman’s single-factor test was initially conducted. However, we acknowledge that this method alone is widely considered insufficient for fully ruling out CMV. Therefore, we adopted the procedure recommended by Mossholder et al. (34), comparing two models based on differences in degrees of freedom and chi-square values. Model 1 yielded a chi-square value of 5022.571 (df = 495, *p <* 0.001), while Model 2 produced a chi-square of 1034.454 (df = 443, *p <* 0.001). The relative fit between the models indicated no support for a single-factor structure, suggesting that CMV was not a significant concern in this study. Nevertheless, future research should consider employing additional procedural remedies to further mitigate CMV, such as collecting data from multiple sources, separating measurement times, or using different response formats for predictor and criterion variables.

To enhance transparency and replicability, we added the following methodological details: (1) Missing data were minimal (<5%) and handled using listwise deletion; (2) Prior to conducting SEM, we examined key assumptions. Normality was assessed via skewness and kurtosis (all values within acceptable ranges, i.e., |skewness| < 2, |kurtosis| < 7); linearity and multicollinearity were evaluated using residual plots and VIF values (all VIFs < 3); (3) For the mediation analysis, bootstrapping was conducted with 5,000 samples using bias-corrected 95% confidence intervals; (4) The average time to complete the questionnaire was approximately 5–7 min, with a dropout rate of about 5%.

## 3 Results

### 3.1 Measurement model

Confirmatory factor analysis (CFA) was conducted using AMOS v23 to assess the reliability and validity of the latent variables. All constructs demonstrated strong internal consistency, with Cronbach’s *α* values exceeding 0.8 (see Table 3), consistent with Fornell and Larcker (16) threshold. Convergent validity was also supported, as the average variance extracted (AVE) for each construct exceeded 0.5, and composite reliability (CR) values were all above 0.8. Factor loadings ranged from 0.606 to 0.852, falling within the “very good” to “excellent” range based on Comrey and Lee (11) criteria, further confirming the model’s construct validity.

**Table 3:** Reliability and validity.

Items	Factor loadings	Cronbach’s *α*	CR	AVE
Mindfulness (MIN)		0.930	0.926	0.716
MIN1	0.831			
MIN2	0.810			
MIN3	0.845			
MIN4	0.823			
MIN5	0.825			
Perfectionism (PER)		0.932	0.933	0.700
PER1	0.826			
PER2	0.827			
PER3	0.824			
PER4	0.807			
PER5	0.804			
PER6	0.793			
Ego-depletion (ED)		0.903	0.873	0.538
ED1	0.662			
ED2	0.685			
ED3	0.780			
ED4	0.606			
ED5	0.850			
ED6	0.788			
Fear of Failure (FOF)		0.842	0.863	0.613
FUF	0.844			
FIOLS	0.852			
FUIO	0.797			
FESE	0.806			

FUF, Fear of an Uncertain Future; FIOLS, Fear of Important Others’ Losing Interest; FUIO, Fear of Upsetting Important Others; FESE, Fear of Experiencing Shame and/or Embarrassment.

Discriminant validity was established by verifying that the square root of each construct’s AVE (on the diagonal) was greater than its correlations with other constructs (see Table 4), indicating that the constructs were empirically distinct.

**Table 4:** Pearson correlation.

Construct	MIN	PER	ED	FOF
MIN	(0.846)			
PER	−0.508**	(0.837)		
ED	−0.423**	0.448**	(0.733)	
FOF	−0.422**	0.561**	0.504**	(0.783)

The square root of the AVE is in diagonals; off diagonals are a Person’s corrections of contracts. **p < 0.01.

### 3.2 Structural mod**el**

**Figure 2:**
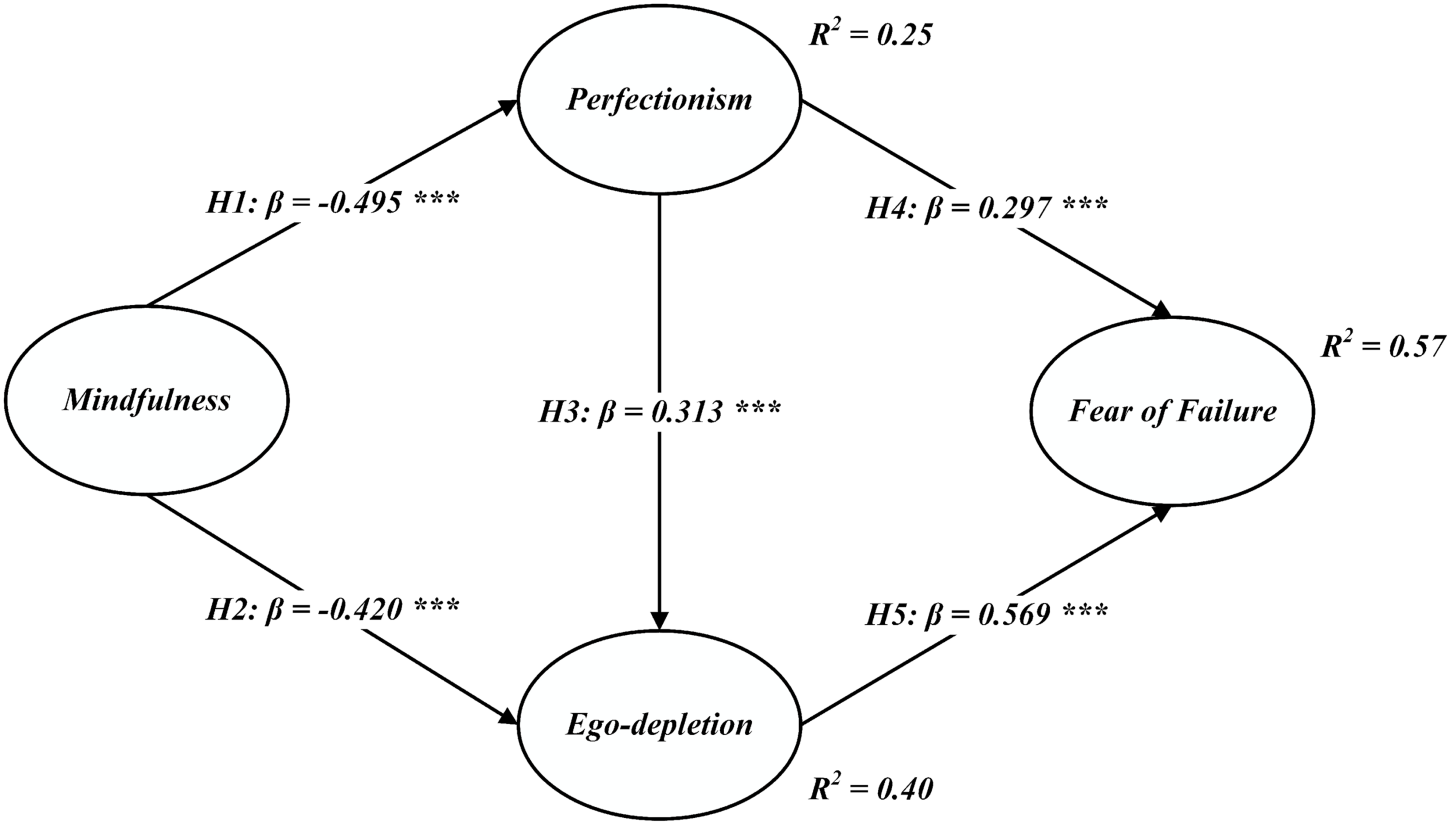
Structural path model. All values represent standardized path coefficients. ***p < 0.001. 95% confidence intervals are provided in Table 5.

Following confirmation of the measurement model’s reliability and validity, the structural model was analyzed using AMOS v23 to test the proposed hypotheses. Results from confirmatory factor analysis (CFA), based on 5,000 bootstrap samples, indicated a good model fit: χ^2^/df = 2.132, GFI = 0.820, NFI = 0.874, TLI = 0.922, CFI = 0.929, and RMSEA = 0.063. These values meet commonly accepted thresholds, supporting the model’s adequacy. Pearson correlation results (see Table 4) further confirmed significant associations among the key variables, while standardized path coefficients are presented in Figure 2.

As shown in Figure 2, mindfulness was negatively associated with perfectionism (*β* = −0.495, *p <* 0.001) and ego-depletion (*β* = −0.420, *p <* 0.001), supporting H1 and H2, respectively. Perfectionism positively predicted ego-depletion (*β* = 0.313, *p <* 0.001), confirming H3. Additionally, perfectionism was positively associated with fear of failure (*β* = 0.279, *p <* 0.01), supporting H4, while ego-depletion also showed a strong positive relationship with fear of failure (*β* = 0.569, *p <* 0.001), confirming H5.

As shown in Table 5, the mediating effects were tested using bootstrap estimation with 5,000 resamples and 95% bias-corrected confidence intervals. The results revealed a significant indirect effect of mindfulness on fear of failure through perfectionism and ego-depletion (indirect effect = −0.466, SE = 0.047, 95% CI = [−0.555, −0.371], *p <* 0.01), providing strong support for H6.

**Table 5:** Standardized direct and indirect effects.

Paths	Point estimate	Product of coefficients		Bootstrapping		
				Bias-corrected 95% CI		Two-tailed significance
		SE	Z	Lower	Upper	
Standardized direct effects						
MIN → PER	−0.495	0.070	−7.072	−0.623	−0.345	*p <* 0.01
MIN → ED	−0.420	0.090	−4.667	−0.586	−0.231	*p <* 0.001
PER→ED	0.313	0.096	3.260	0.135	0.511	*p <* 0.01
PER→FOF	0.279	0.113	2.469	0.077	0.529	*p <* 0.01
ED → FOF	0.569	0.099	5.747	0.349	0.738	*p <* 0.001
Standardized indirect effects						
MIN → FOF	−0.466	0.047	−9.915	−0.555	−0.371	*p <* 0.01

## 4 Discussion

### 4.1 Theoretical contributions

This study advances the theoretical understanding of athletes’ fear of failure by addressing a critical gap in the existing literature. To date, research on fear of failure has primarily focused on academic and business contexts (10, 20, 52). For example, Choi (10) examined how fear of failure influences individual behavior and performance in learning environments, while Cacciotti et al. (8) explored its dual role as both a motivator and inhibitor in entrepreneurial activities. In the field of sports, however, researchers have largely examined fear of failure in relation to variables such as shame (24), anxiety (22), burnout (24), and aggression (39), focusing primarily on its effects and consequences. Yet, the underlying mechanisms contributing to fear of failure among athletes remain underexplored.

To address this gap, the present study introduces mindfulness, perfectionism, and ego-depletion as key constructs for investigating the potential pathways underlying athletes’ fear of failure. By integrating the constructs of perfectionism and ego-depletion, this study offers a more nuanced and targeted analysis, thereby enriching the theoretical discourse within the sports psychology domain. The findings support the notion that perfectionism contributes significantly to fear of failure among athletes (47), and suggest that addressing this relationship is key to understanding performance breakdowns and developing effective intervention strategies. However, building upon prior research, this study highlights that perfectionistic athletes often impose excessively high standards on themselves and engage in harsh self-criticism during competitions. Studies have shown that such athletes tend to experience higher levels of fear, anxiety, and stress in competitive settings (35). Under such circumstances, athletes are forced into continuous self-regulation and behavioral control to maintain competitive performance (30). As their psychological resources for self-regulation become gradually depleted, they are more likely to enter a state of ego-depletion, which undermines their capacity for further self-control and exacerbates anxiety and psychological strain (28). The findings of this study further indicate that athletes with higher levels of ego-depletion also exhibit correspondingly higher levels of fear of failure. Prolonged exposure to this negative cycle may lead athletes to develop various psychological and somatic disorders, severely compromising their mental health and hindering their athletic careers.

Second, this study is the first to explore the relationship between mindfulness and fear of failure through the mediating roles of perfectionism and ego-depletion. The findings revealed that mindfulness was significantly negatively associated with both perfectionism and ego-depletion; perfectionism positively predicted ego-depletion; and both perfectionism and ego-depletion were positively associated with fear of failure (see Figure 2). These results are largely consistent with previous studies. For example, Appleton et al. (3) found that multidimensional perfectionism was positively associated with athlete burnout; Yusainy and Lawrence (54) reported that mindfulness was negatively associated with aggressive behavior, fear, and anger; and Tobin and Dunkley (48) indicated a negative interaction between self-critical perfectionism and mindfulness. Notably, ego-depletion emerged as the strongest predictor of fear of failure, followed by the negative effect of mindfulness on perfectionism. Mediation analysis confirmed that perfectionism and ego-depletion jointly mediated the link between mindfulness and fear of failure. The model accounted for 57% of the variance in fear of failure, offering a novel explanatory pathway: mindfulness mitigates the psychological cost of self-control, enabling individuals to better manage perfectionistic tendencies and reduce fear of failure—particularly when supported by mindfulness-based interventions.

### 4.2 Practical implications

Given the potential roles of perfectionism, ego-depletion, and fear of failure, as well as the findings of this study—which suggest that individuals with higher levels of mindfulness tend to report lower levels of perfectionism, ego-depletion, and fear of failure—it is recommended that both athletes and coaches incorporate mindfulness meditation into their daily training routines, supported by adequate material resources and psychological guidance.

For athletes, engaging in mindfulness meditation can help them better regulate their emotions, focus attention on the present moment, and reduce distractions caused by other psychological factors such as perfectionism, anxiety, and fear of failure (51). Moreover, mindfulness training encourages individuals to be present and to acknowledge their current experiences, which may help athletes recognize and accept their limitations, thereby mitigating the negative effects of setting excessively high goals and engaging in harsh self-criticism (48). Additionally, coaches and family members should adjust their attitudes, prioritizing encouragement and support. They should guide athletes not only technically but also pay close attention to their emotional and psychological states, especially after setbacks, thereby reducing the psychological harm caused by failure (33).

In addition, this study examined the general status of fear of failure among athletes and its potential consequences, while highlighting mindfulness training as an effective intervention to reduce such fear. By exploring fear of failure through the lenses of perfectionism and ego-depletion, the study deepens athletes’ understanding of this emotion. It encourages a shift in perception—from viewing fear of failure solely as shameful and negative, to recognizing it as an objective experience that can, to some extent, be constructive.

For a long time, fear has been misunderstood and often associated with negative personality traits such as cowardice or weakness. Athletes, however, are typically expected to embody qualities such as resilience and physical strength, and are often seen as exemplars of positive characteristics (1). As a result, many athletes may harbor a degree of aversion toward their own fear-related emotions, which can hinder them from confronting these feelings or seeking help when their fear of failure becomes excessive. This reluctance may ultimately lead to psychological difficulties, hinder athletic performance, and in some cases, even contribute to the premature end of their sports careers.

### 4.3 Limitations

This study has several limitations. First, the use of snowball and convenience sampling, with data primarily collected from athletes in Hunan, Hubei, and Sichuan provinces, may limit the generalizability of the findings due to the relatively narrow geographic coverage. Although this approach was necessary due to time and resource constraints, future studies should conduct broader, nationwide data collection to improve the representativeness and robustness of the results.

Second, the study did not address the cultural validity of the Western-developed instruments used in the Chinese context. Cultural factors may shape how participants understand and respond to certain items, potentially affecting the reliability and interpretation of the results. Future research should consider cultural adaptation and validation of the instruments to ensure the cross-cultural applicability and conceptual clarity of the constructs.

Third, the scores of the Depletion Sensitivity Scale (DSS) cannot fully reflect individuals’ current level of ego-depletion or its momentary fluctuations. Rather, they only indicate that individuals with higher depletion sensitivity are more likely to enter a state of ego-depletion under resource-consuming conditions. The present study therefore does not focus on the actual level of ego-depletion or its dynamic fluctuations, but instead emphasizes the stable individual differences associated with depletion sensitivity and their relationship with fear of failure. Future research should adopt multidimensional measurement tools to provide a more comprehensive examination of the links between ego-depletion and fear of failure.

Fourth, the 5-item Mindful Attention Awareness Scale (MAAS-5) may not fully capture the multifaceted nature of mindfulness. While it measures attention and awareness, it does not address other key components such as acceptance and non-reactivity. Furthermore, although the Depletion Sensitivity Scale (DSS) showed acceptable composite reliability, its AVE was borderline at 0.538, suggesting that a substantial portion of item variance may be due to measurement error. Future studies are encouraged to adopt more comprehensive and multidimensional instruments to enhance the validity of these constructs.

## 5 Conclusion

In addressing the research objectives, this study underscores that failure is an inevitable part of every athlete’s career, often accompanied by significant psychological distress. Regulating fear of failure and perfectionistic self-criticism demands substantial psychological resources; once these resources are depleted, athletes’ capacity for self-regulation declines, increasing the likelihood of performance breakdowns and failure. The findings identify mindfulness, perfectionism, and ego-depletion as key factors influencing this process. Specifically, individuals with higher levels of mindfulness tend to exhibit lower levels of perfectionism, ego-depletion, and fear of failure.

Based on these insights, it is recommended that athletes and coaches integrate mindfulness meditation into daily training routines, supported by appropriate material and psychological resources. Coaches and family members should also adjust their attitudes, offering encouragement to athletes, helping them develop a healthier perspective on failure, and paying greater attention to their emotional and psychological states, especially when they encounter setbacks, in order to minimize the psychological harm caused by failure. Additionally, athletes should learn to accept and acknowledge their fear of failure, and when struggling with perfectionism or fear of failure, they should seek professional assistance in a timely manner to ensure both their physical and mental well-being.

## Data Availability

The raw data supporting the conclusions of this article will be made available by the authors, without undue reservation.
